# The presence and severity of cerebral small vessel disease increases the frequency of stroke in a cohort of patients with large artery occlusive disease

**DOI:** 10.1371/journal.pone.0184944

**Published:** 2017-10-09

**Authors:** Ki-Woong Nam, Hyung-Min Kwon, Jae-Sung Lim, Moon-Ku Han, Hyunwoo Nam, Yong-Seok Lee

**Affiliations:** 1 Department of Neurology, Seoul National University College of Medicine, Seoul, Korea; 2 Department of Neurology, Seoul Metropolitan Government-Seoul National University Boramae Medical Center, Seoul, Korea; 3 Department of Neurology, Hallym University Sacred Heart Hospital, Anyang, Korea; 4 Department of Neurology, Seoul National University Bundang Hospital, Seongnam, Korea; Massachusetts General Hospital, UNITED STATES

## Abstract

**Background:**

Cerebral small vessel disease (SVD) commonly coexists with large artery atherosclerosis (LAA).

**Aim:**

We evaluate the effect of SVD on stroke recurrence in patients for ischemic stroke with LAA.

**Methods:**

We consecutively collected first-ever ischemic stroke patients who were classified as LAA mechanism between Jan 2010 and Dec 2013. Univariate and multivariate Cox analyses were performed to evaluate the association between the 2-year recurrence and demographic, clinical, and radiological factors. To evaluate the impact of SVD and its components on recurrent stroke, we used the Kaplan-Meier analysis. SVD was defined as the presence of severe white matter hyperintensity (WMH) or old lacunar infarction (OLI) or cerebral microbleeds (CMB). We also compared frequency and burden of SVD among recurrent stroke groups with different mechanisms.

**Results:**

Among a total of 956 participants, 92 patients had recurrent events. Recurrence group showed a higher frequency of severe WMH, OLI, asymptomatic territorial infarction, and severe stenosis on the relevant vessel in multivariate analysis. The impact of SVD and its components on recurrent stroke was significant in any ischemic recurrent stroke, and the presence of SVD was continuously important in stroke recurrence regardless of its mechanism, including recurrent LAA stroke, recurrent small vessel occlusion stroke, and even recurrent cardioembolic stroke. Additionally, the recurrence rate increased in dose-response manner with the increased number of SVD components.

**Conclusions:**

Cerebral SVD is associated with recurrent stroke in patients with LAA. Additionally, it may affect any mechanisms of recurrent stroke and even with a dose response manner.

## Introduction

Cerebral small vessel disease (SVD) commonly coexists with large artery atherosclerosis (LAA), and ischemic stroke from LAA has a high risk of stroke recurrence, especially with intracranial atherosclerosis. [1] Although LAA commonly coexists with cerebral SVD and shares many risk factors with SVD, [2–5] these two entities of cerebral vessel disease are thought to had different pathophysiologic mechanisms. [6, 7] Furthermore, the clinical impact of SVD on stroke recurrence in patients with LAA has not been well investigated.

Recently, the study from the Stenting and Aggressive Medical Management for Preventing Recurrent Stroke in Intracranial Stenosis (SAMMPRIS) trial showed a high frequency of SVD in patients with severe intracranial atherosclerosis (ICAS) with marginal clinical significance on stroke recurrence. [8] However, due to the small number of included patients and traits of post-hoc analysis, the study was limited in proving the effect of SVD on stroke recurrence in patients with intracranial atherosclerosis. In this study, we aimed to evaluate the association between SVD and 2-year stroke recurrence after first-ever ischemic stroke with LAA in a large number of patients. In addition, we also assessed the mechanisms of recurrent stroke to investigate clinical impact of SVD on stroke recurrence in this population.

## Materials and methods

### Ethics statement

This study was approved by the institutional review board (IRB) at Seoul Metropolitan Government-Seoul National University Boramae Medical Center (IRB No.16-2016-120). This study was designed as a retrospective study in which medical records were only reviewed. Thus, informed consent was not needed and even unattainable. Understanding of this problem, the IRB of Seoul Metropolitan Government-Seoul National University Boramae Medical Center approved this study, despite not having informed consent.

### Patients and population

**Fig 1:**
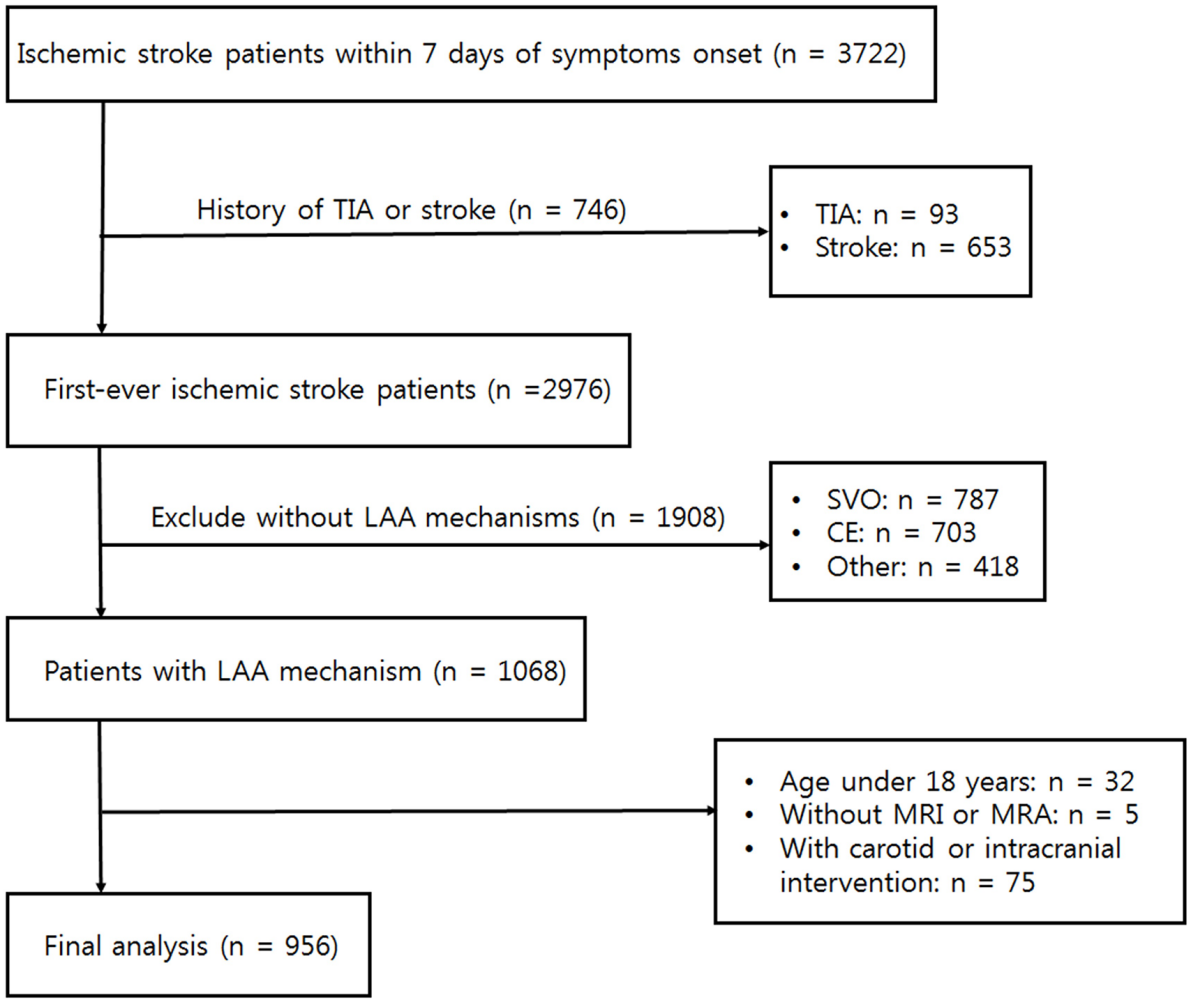
Patient selection flow chart.

We initially collected consecutive first-ever acute ischemic stroke patients who visited the Seoul Metropolitan Government-Seoul National University Boramae Medical Center and Seoul National University Bundang Hospital within 7 days of symptom onset between January. 2010 and December. 2013 (n = 2976). We excluded the recurrent stroke patients since they showed higher stroke recurrence than the first-ever stroke patients and had a different disease condition. Then, we extracted the subpopulation classified as LAA (more than 50% stenosis or occlusion of relevant artery) using the Trial of Org 10172 in Acute Stroke Treatment (TOAST) classification (n = 1068). [9] Patients with two or more or other etiology mechanisms (e.g., vasculitis, arterial dissection, hypercoagulability, and hematologic disease) were not included (n = 418). Patients who met the following criteria were also excluded: being under 18 years of age (n = 32), or not having brain magnetic resonance imaging (MRI) or magnetic resonance angiography (MRA) performed on them (n = 5). We excluded patients who underwent intervention (e.g. carotid endarterectomy, carotid artery stenting, intracranial artery stenting) to limit their natural course of diseases with medical treatment. (n = 75). Finally, a total of 956 participants remained following the final analysis (Fig 1).

### Clinical assessment

We evaluated all the demographic factors, the cardiovascular risk factors of stroke, and clinical factors including age, sex, hypertension, diabetes, hypercholesterolemia, current smoking, use of alcohol, severity of stroke, initial systolic/diastolic blood pressure, use of thrombolytic therapy, and discharge treatment. Severity of stroke was assessed by well-trained neurologists using the National Institutes of Health Stroke Scale (NIHSS) score at the time of admission. [10] Discharge treatment was divided into three categories: anti-thrombotic agents, anti-hypertensives, and statins. Anti-thrombotic agents were subdivided into mono anti-platelet agent and dual anti-platelet agent.

The primary outcome in the study was a 2-year recurrence of any ischemic stroke. We defined the recurrence when a new clinical event accompanied with a new brain lesion on MRI, being spatially distinct from the index stroke. The survival time was defined as the time to the recurred event or 2-year complete follow-up. The survival time in patients who had multiple recurrent events was defined as the time to the first recurred event. The evaluation of recurrence and the duration of survival time were performed by two investigators who were blinded to other clinical and radiological information about the index stroke. The mechanism of recurrent stroke was defined using the TOAST classification. Recurrent LAA stroke was dichotomized into the recurrent stroke in the same territory and the recurrent stroke in the different territory.

### Radiological assessment

All patients underwent brain MRI and MRA using 1.5-Tesla or 3.0-Tesla MR scanners (Achieva 1.5T and 3.0T; Philips, Amsterdam, the Netherlands) within 24 hours of admission, recording T1-weighted images [repetition time (TR)/echo time (TE) = 300/10 or 500/10], T2-weighted images (TR/TE = 3000/100 or 5100/90), diffusion-weighted images (DWI) (TR/TE = 4800/66 or 3000/44), fluid attenuated inversion recovery images (TR/TE = 10000/90), T2-gradient echo images (TR/TE = 28/20 or 57/20), and three-dimensional time of flight MRA images (TR/TE = 24/3.5 or 20/7, slice of thickness = 1.2mm). The underlying SVD was assessed in three aspects: white matter hyperintensity (WMH), old lacunar infarction (OLI), and cerebral microbleeds (CMB). WMH was rated using the Fazekas scale both in periventricular and subcortical areas. [11] We then summed up their score and dichotomized into mild (0–2) or severe (3–6) WMH. [12] OLI was defined as the size of 3–15 mm of well-defined lesions on perforator areas with the same signal characteristics as cerebrospinal fluid on MRI. [13] We defined CMB as a focal round area of low signal on T2-gradient echo images of less than 10 mm in diameter. [14] CMB was counted in lobar and deep/infratentorial areas, respectively, considering their different pathophysiology. [15] We then defined the SVD, in this study, as the presence of severe WMH, or OLI, or CMB. [8] Asymptomatic territorial infarction is an old infarction in the territory of relevant artery apart from the lacunar areas. The relevant vessel locations were categorized as intracranial or extracranial. The severity of stenosis on the relevant vessel was dichotomized into either severe (≥ 70%) or moderate (50–69%). The radiological findings were rated by two neurologists without clinical information, showing the mean inter-rater reliability coefficients of *P* = 0.892.

### Statistical analysis

Univariate Cox analysis was used to evaluate the relationship between demographic, clinical, or radiological factors and the 2-year recurrent stroke, being adjusted by the time to primary end point (survival time). All variables of *P* < 0.05 from the univariate analysis were introduced into the multivariate Cox regression analysis as confounders. To clarify the impact of SVD on 2-year stroke recurrence, we conducted the Kaplan-Meier analysis using SVD and any 2-year recurrent stroke. Then, the components of SVD (e.g., severe WMH, OLI, or CMB) were respectively introduced, replacing the SVD variable, to evaluate the impact of each component.

We also evaluated the baseline characteristics with and without SVD. Continuous variables were assessed by the Student’s *t*-test or the Mann-Whitney *U*-test, and we used chi-squared test or Fisher’s exact test for the categorical variables. Continuous variables with skewed data were transformed into a log scale. To evaluate the effect of burden of SVD on the recurrent stroke mechanism, we compared presence of severe WMH, OLI, CMB and the number of components of SVD among three groups [e.g. LAA, small vessel occlusion (SVO), and cardioembolism (CE)]. We used the Kruskal-Wallis test for continuous variables and chi-squared or Fisher’s exact test for the categorical variables. Variables with *P*-value < 0.05 were considered significant. All statistical analyses were performed using SPSS version 21 (IBM, SPSS, Chicago, IL, USA).

## Results

We included a total of 956 participants (mean age 66 years, median NIHSS score 3 [1–6]). The mean visit time after symptoms onset was 46 hours, and 50% of them visited within 24 hours. The follow-up durations of the cohort was 2.8 [1.4–4.1] years, and about 69% of them had completed a 2-year follow-up (80% of them had completed a 1-year follow-up). We found 92 patients who had recurrent stroke within 2-years, and the recurrence rate adjusted by the survival time using the Kaplan-Meier analysis was 10.6%.

Baseline characteristics of the demographic, clinical, and radiological factors with and without recurrence are shown in Table 1. The recurrence group had older age, higher frequencies of severe WMH, CMB, OLI, asymptomatic territorial infarction, and severe stenosis of the relevant artery. They also showed marginal trends of more frequent use of dual anti-platelet agent without statistical significance.

**Table 1:** Baseline characteristics between with and without a 2-year stroke recurrence.

	No recurrence (n = 864)	Recurrence (n = 92)	HR (95% CI)	*P*
Age, y [IQR]	67 [57–75]	71 [60–79]	1.03 [1.01–1.04]	0.006
Sex, male %	551 (64)	62 (67)	1.14 [0.74–1.76]	0.562
Hypertension, %	607 (70)	66 (72)	1.09 [0.69–1.71]	0.719
Diabetes, %	312 (36)	32 (35)	0.97 [0.63–1.50]	0.901
Hyperlipidemia, %	251 (29)	28 (30)	1.02 [0.65–1.58]	0.944
Current smoking, %	294 (34)	23 (25)	0.64 [0.40–1.03]	0.067
Alcohol, %	365 (42)	37 (40)	0.90 [0.59–1.37]	0.623
Initial NIHSS [IQR]	3 [1–6]	4 [2–5]	1.02 [0.98–1.06]	0.325
SBP, mmHg [IQR]	155 [140–171]	150 [139–170]	1.00 [0.99–1.01]	0.584
DBP, mmHg [IQR]	84 [75–94]	83 [74–94]	1.00 [0.98–1.01]	0.585
Thrombolysis, %			1.14 [0.79–1.64]	0.489
None	802 (93)	85 (92)		
Intravenous	29 (3)	1 (1)		
Intraarterial	20 (2)	5 (5)		
Both	13 (2)	1 (1)		
Discharge treatment, %			1.52 [0.99–2.33]	0.058
Mono anti-platelet agent	393 (46)	32 (35)		
Dual anti-platelet agent	468 (54)	60 (65)		
Anti-hypertensive, %	559 (65)	54 (59)	0.78 [0.51–1.19]	0.243
Statin, %	745 (87)	81 (88)	1.08 [0.57–2.02]	0.815
Severe WMH, %	154 (18)	39 (42)	3.29 [2.18–4.98]	< 0.001
Cerebral microbleeds, %	182 (21)	33 (36)	2.00 [1.31–3.06]	0.001
Old lacunar infarction, %	267 (31)	54 (59)	3.05 [2.02–4.62]	< 0.001
Asymptomatic territorial infarction, %	103 (12)	26 (28)	2.82 [1.79–4.43]	< 0.001
Relevant vessel location, %			1.34 [0.88–2.02]	0.173
Intracranial	569 (66)	54 (59)		
Extracranial	295 (34)	38 (41)		
Severe stenosis, %	402 (47)	60 (65)	2.15 [1.40–3.30]	< 0.001

NIHSS = National Institutes of Health Stroke Scale, SBP = systolic blood pressure, DBP = diastolic blood pressure, WMH = white matter hyperintensity

In the multivariate analysis using Cox regression analysis, severe WMH [adjusted OR (aOR) = 2.23, 95% confidence interval (CI) = 1.35–3.70, *P* = 0.002], OLI [aOR = 2.24, 95% CI = 1.43–3.53, *P* < 0.001], asymptomatic territorial infarction [aOR = 1.89, 95% CI = 1.18–3.03, *P* = 0.008], and severe stenosis [aOR = 2.41, 95% CI = 1.56–3.72, *P* < 0.001] remained significant after adjusting confounders (Table 2). These results continued after we additionally adjusted discharge treatment options. (S1 Table)

**Table 2:** Multivariate analysis of possible predictors of 2-year stroke recurrence.

	Crude OR	*P*	Adjusted OR	*P*
Age	1.03 [1.01–1.04]	0.006	1.00 [0.99–1.02]	0.675
Severe white matter hyperintensity	3.29 [2.18–4.98]	< 0.001	2.23 [1.35–3.70]	0.002
Old lacunar infarction	3.05 [2.02–4.62]	< 0.001	2.24 [1.43–3.53]	< 0.001
Asymptomatic territorial infarction	2.82 [1.79–4.43]	< 0.001	1.89 [1.18–3.03]	0.008
Cerebral microbleeds	2.00 [1.31–3.06]	0.001	1.14 [0.71–1.84]	0.585
Severe stenosis	2.15 [1.40–3.30]	< 0.001	2.41 [1.56–3.72]	< 0.001

We used binary logistic regression analysis adjusted by age, severe white matter hyperintensity, old lacunar infarction, asymptomatic territorial infarction, cerebral microbleeds, and severe stenosis of relevant artery

**Fig 2:**
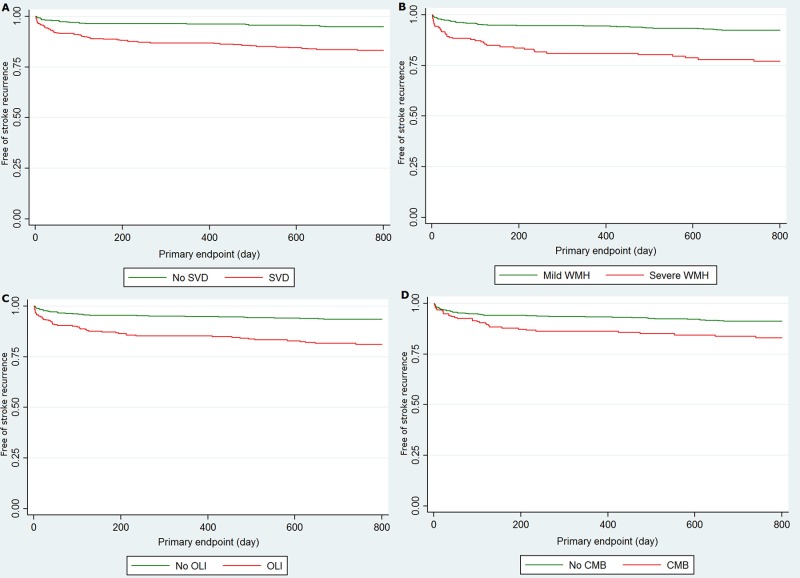
Recurrent stroke between with and without SVD, severe WMH, OLI, or CMB. Recurrent stroke rate was significantly higher in the group with small vessel disease (A) (*P* < 0.001), severe white matter hyperintensity (B) (*P* < 0.001), old lacunar infarction (C) (*P* < 0.001), or cerebral microbleeds (D) (*P* < 0.001).

**Fig 3:**
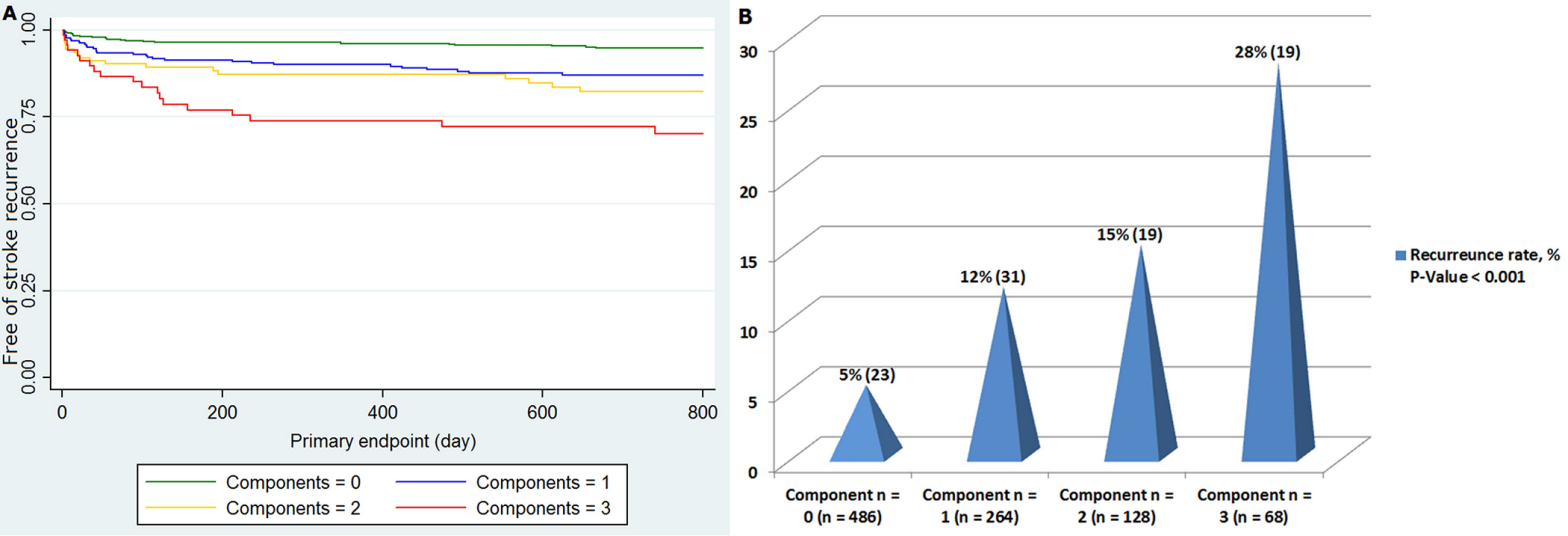
Recurrent stroke with the number of components of small vessel disease. Number of components of small vessel disease showed a dose-response manner with 2-year recurrent stroke both in the Kaplan-Meier analysis (*P* < 0.001) (A) and univariate Cox regression analysis adjusted by survival time (*P* < 0.001) (B).

The impact of SVD on recurrent stroke was analyzed using the Kaplan-Meier analysis (Fig 2). The rate of recurrence was significantly different not only with the presence of SVD (log rank test, *P* < 0.001), but also with severe WMH, OLI, or CMB, respectively (log rank test, *P* < 0.001 and *P* = 0.001, respectively). We found SVD in a total of 460 patients, and participants with SVD had higher age, were more frequently female, had more frequent hypertension, and an intracranial index stroke (Table 3). Among the 460 SVD patients, 264 (57%) had one component of SVD (42 WMH, 152 OLI, and 70 CMB), remaining 128 (28%) had two (52 WMH and OLI, 28 WMH and CMB, and 48 OLI and CMB) and 68 (15%) had three components. Additionally, the number of components of SVD and recurrent stroke showed a dose-response manner (Fig 3).

**Table 3:** Baseline characteristics between with and without small vessel disease.

	No SVD (n = 486)	SVD (n = 460)	*P*
Age, y [IQR]	63 [54–71]	72 [63–79]	< 0.001
Sex, male %	334 (69)	271 (59)	0.002
Hypertension, %	301 (62)	363 (79)	< 0.001
Diabetes, %	168 (35)	172 (37)	0.366
Hyperlipidemia, %	147 (30)	128 (28)	0.412
Initial NIHSS [IQR]	3 [1–5]	3 [2–6]	0.110
SBP, mmHg [IQR]	154 [140–172]	155 [140–171]	0.795
DBP, mmHg [IQR]	85 [76–95]	83 [74–93]	0.173
Relevant vessel location, %			0.002
Intracranial	294 (60)	322 (70)	
Extracranial	192 (40)	138 (30)	
Severe stenosis, %	244 (50)	213 (46)	0.230
Recurrent stroke mechanism, %			0.212
Large artery atherosclerosis	20 (87)	49 (71)	
Small vessel occlusion	1 (4)	11 (16)	
Cardioembolism	2 (9)	8 (12)	
Other	0 (0)	1 (1)	

SVD = small vessel disease, NIHSS = National Institutes of Health Stroke Scale, SBP = systolic blood pressure, DBP = diastolic blood pressur

**Fig 4:**
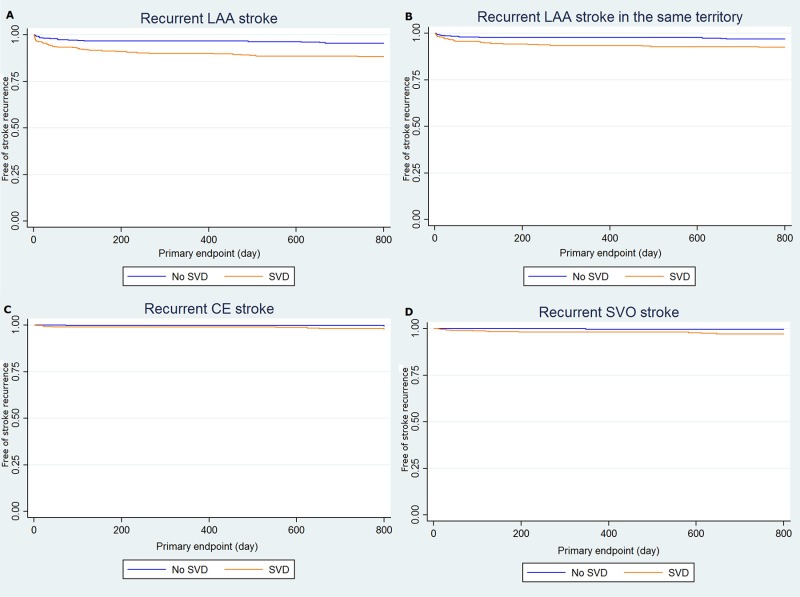
Effect of small vessel disease on stroke recurrence in different recurrence mechanisms. Patients with small vessel disease had higher chances of recurrent large artery atherosclerosis stroke (A) (*P* < 0.001), recurrent large artery atherosclerosis stroke in the same territory of the index stroke (B) (*P* = 0.003), recurrent cardioembolism stroke (C) (*P* = 0.033), and recurrent small vessel occlusion stroke (D) (*P* = 0.002). Small vessel disease could be a risk factor of recurrent stroke without considering its recurrence mechanisms.

In a total of 92 recurrent strokes, 69 patients were classified with the LAA mechanism, remaining 12 SVO, 10 CE, and 1 other (e.g. essential thrombocytosis). Among the 69 LAA recurrent strokes, 45 patients had a recurrent stroke in the same territory. To evaluate the effect of SVD on the recurrent stroke mechanism, we compared the frequency and burden of SVD among recurrent LAA, SVO, and CE patients (S2 Table, S1 Fig). There were no significant differences about burden of SVD among different mechanisms, however, patients with SVD had higher chances of recurrent stroke than without SVD regardless of its recurrence mechanism (Fig 4).

## Discussion

In this study, we found that SVD and its components (severe WMH, OLI, and CMB) were associated with the recurrent stroke in patients with LAA. Additionally, these findings affected not only recurrent SVO stroke, known to share the same pathophysiologic mechanism of SVD, [6, 7] but the recurrent stroke with other mechanisms (recurrent LAA stroke in the same territory, recurrent LAA stroke in the different territory, and recurrent CE stroke). The rate of recurrent stroke also increased when the number of SVD components increased in a dose-response manner.

We found that more than half of the recurrent strokes resulted from the relevant artery of the index stroke in patients with LAA. Interestingly, the recurrence from the relevant large artery was also affected by the SVD. There are several possible explanations about the effect of SVD on the LAA. First, severe SVD may imply a hostile brain environment which is vulnerable to ischemic insult. In patients with SVD, diffuse hypoperfusion and long-standing hypertension, which commonly leads to lipohyalinosis, are most well-known causes of it. [16, 17] In this condition, cerebral auto-regulation and compensatory collateral blood flow are impaired. [18, 19] Thus, a minor ischemic insult could cause symptomatic stroke events. Second, SVD itself could be an evidence of recurrent stroke. There have been several studies showing OLI or severe WHM are sequelae of previous asymptomatic stroke events. [20, 21] Thus, patients with higher burden of OLI or WMH may experience multiple stroke events, and may have a higher chance for further recurrence. Third, SVD might be the result of intracranial atherosclerosis which is a well-known predictor of stroke recurrences. Considering its anatomical location, intracranial atherosclerosis could easily occlude the origin of perforating artery and frequently coexist with SVD. As our study presented, patients with SVD may have higher frequencies of intracranial atherosclerosis and be in high risk of stroke recurrence.

Previously, SVD was argued on whether it represents a simple intermediate surrogate of cardiovascular risk factors or is an independent risk factor of stroke by itself. [4] However, in this study, patients with SVD showed no differences in risk factors except for age and hypertension compared with the non-SVD group, and remained significant after adjusting for confounders. Thus, we thought that SVD by itself might not be a marker of poorly controlled risk factors, but that it may be a predictor of poor prognosis. Furthermore, 43% of patients with SVD in our study showed more than 2 components of SVD and there was a dose-response relationship between the number of components of SVD and the 2-year stroke recurrence rate. Thus, we thought that participants who had multiple components of SVD had a larger burden of SVD and had a higher chance of stroke recurrence.

In this study, patients with SVD had older age, and frequent hypertension, being same traits as previous study which used same the same definition of SVD [8]. Without differences of baseline characteristics, however, they showed much higher 2-year recurrent ischemic stroke rate in both with and without SVD groups with marginal statistical significance of SVD. It may result from small sample size, since they used only 149 participants in analysis of recurrence. Second, the SAMMPRIS trial included only patients with ICAS, which is well-known risk factor of stroke recurrence. Thus, ICAS may strongly affect the subsequent recurrence in both with and without SVD group, and the effects of SVD would be interrupted, showing also high recurrence rate in SVD (-) group. Interestingly, being different from the previous study, female patients presented frequent SVD in our cohort. However, ICAS was also frequent in females, and this statistical significance was removed after adjusting confounders of SVD, including ICAS. Thus, it would be coincidence of frequent ICAS lesions.

We have several caveats in this study. First, it was a two-center retrospective study. Selection bias is possible, and generalization of the findings to a non-Asian population having less frequent SVD should be cautious. Second, we defined the stroke recurrence in terms of requiring both clinical and radiological features. The rate of stroke recurrence might be reduced, compared with previous studies having only clinical definitions. [22, 23] Third, we included patients within 7 days from their symptoms onset, having the possibility of missing early recurrences. However, 50% of participants visited within 24 hours, and 79% within 72 hours. Thus, the effects of the visit time may not change the results.

In conclusion, cerebral SVD is associated with stroke recurrence in ischemic stroke patients with LAA in a dose-response manner. Additionally, in these patients, SVD could affect stroke recurrence regardless of its mechanism.

## Supporting information

S1 Fig Burden and distribution of small vessel disease between recurrent stroke with different mechanisms. Proportions of small vessel disease and distributions about its components were not significantly different among different recurrent mechanisms (A). Burden of small vessel disease, representing by the number of its components, also showed no statistical differences among three groups (B).
(EPS)

S1 Table Multivariate analysis of possible predictors of 2-year stroke recurrence including discharge treatment. (DOCX)

S2 Table Burden of small vessel disease between different mechanisms of recurrent stroke. (DOCX)
