# Supplementary material for: The presence and severity of cerebral small vessel disease increases the frequency of stroke in a cohort of patients with large artery occlusive disease
Source: PLoS One. 2017 Oct 9;12(10):e0184944. doi: 10.1371/journal.pone.0184944 (PMC5633141; doi:10.1371/journal.pone.0184944)
Supplement: S1 Table — (DOCX) [file pone.0184944.s002.docx]

**S1 Table. Multivariate analysis of possible predictors of 2-year stroke recurrence including discharge treatment**

|  | Crude OR | *P-*value | Adjusted OR | *P* value |
| --- | --- | --- | --- | --- |
| Age | 1.03 [1.01-1.04] | 0.006 | 1.01 [0.99-1.03] | 0.622 |
| Severe white matter hyperintensity | 3.29 [2.18-4.98] | < 0.001 | 2.24 [1.36-3.70] | 0.002 |
| Old lacunar infarction | 3.05 [2.02-4.62] | < 0.001 | 2.21 [1.41-3.48] | 0.001 |
| Asymptomatic territorial infarction | 2.82 [1.79-4.43] | < 0.001 | 1.86 [1.16-2.98] | 0.010 |
| Cerebral microbleeds | 2.00 [1.31-3.06] | 0.001 | 1.14 [0.71-1.84] | 0.587 |
| Severe stenosis | 2.15 [1.40-3.30] | < 0.001 | 2.34 [1.52-3.62] | < 0.001 |
| Discharge treatment |  |  |  |  |
| Mono anti-platelet agent | Ref | Ref | Ref | Ref |
| Dual anti-platelet agent | 1.52 [0.99-2.33] | 0.058 | 1.41 [0.91-2.17] | 0.122 |

We used binary logistic regression adjusted by age, severe white matter hyperintensity, old lacunar infarction, asymptomatic territorial infarction, cerebral microbleeds, severe stenosis, and discharge treatment.
