# Supplementary material for: The presence and severity of cerebral small vessel disease increases the frequency of stroke in a cohort of patients with large artery occlusive disease
Source: PLoS One. 2017 Oct 9;12(10):e0184944. doi: 10.1371/journal.pone.0184944 (PMC5633141; doi:10.1371/journal.pone.0184944)
Supplement: S2 Table — (DOCX) [file pone.0184944.s003.docx]

**S2 Table. Burden of small vessel disease between different mechanisms of recurrent stroke**

|  | LAA  (n = 69) | SVO  (n = 12) | CE  (n = 10) | *P* value |
| --- | --- | --- | --- | --- |
| Age, y [IQR] | 72 [59-79] | 73 [64-79] | 71 [61-77] | 0.921 |
| Sex, male % | 47 (68) | 6 (50) | 8 (80) | 0.356 |
| Relevant vessel location, % |  |  |  | 1.000 |
| Intracranial | 40 (58) | 7 (58) | 6 (60) |  |
| Extracranial | 29 (42) | 5 (42) | 4 (40) |  |
| Severe white matter hyperintensity | 30 (43) | 6 (50) | 3 (30) | 0.630 |
| Old lacunar infarction | 39 (57) | 10 (83) | 4 (40) | 0.099 |
| Asymptomatic territorial infarction | 16 (23) | 6 (50) | 3 (30) | 0.143 |
| Cerebral microbleeds | 22 (32) | 6 (50) | 5 (50) | 0.323 |
| Number of small vessel disease components |  |  |  | 0.413 |
| 0 | 20 (29) | 1 (8) | 2 (20) | 0.328 |
| 1 | 22 (32) | 3 (25) | 5 (50) | 0.451 |
| 2 | 12 (17) | 5 (42) | 2 (20) | 0.183 |
| 3 | 15 (22) | 3 (25) | 1 (10) | 0.753 |

LAA = large artery atherosclerosis, SVO = small vessel occlusion, CE = cardioembolism
